# Global trends and regional differences in the burden of cancer attributable to secondhand smoke in 204 countries and territories, 1990–2019

**DOI:** 10.3389/fonc.2022.972627

**Published:** 2022-10-11

**Authors:** Mailikezhati Maimaitiming, Minmin Wang, Yanan Luo, Jia Wang, Yinzi Jin, Zhi-Jie Zheng

**Affiliations:** ^1^ Department of Global Health, School of Public Health, Peking University, Beijing, China; ^2^ Institute for Global Health and Development, Peking University, Beijing, China; ^3^ Key laboratory of Carcinogenesis and Translational Research (Ministry of Education/Beijing), Laboratory of Genetics, Peking University Cancer Hospital & Institute, Beijing, China; ^4^ Key Laboratory of Carcinogenesis and Translational Research (Ministry of Education/Beijing), Department of Thoracic Surgery II, Peking University Cancer Hospital & Institute, Beijing, China

**Keywords:** disease burden, cancer, secondhand smoke, trend, Global Health Data Exchange

## Abstract

**Background:**

To describe the status quo and trends in the global burden of all cancers caused by secondhand smoke during 1990–2019.

**Methods:**

Data on cancer associated with secondhand smoke were extracted from the Global Heath Data Exchange. Cancer burden was measured by cancer-related deaths, disability-adjusted life years (DALYs), years lived with disability (YLDs), and years of life lost (YLLs).

**Results:**

In 2019, age-standardized rates of death, DALYs and YLLs among the cancer population globally caused by secondhand smoke were 1.60, 38.54 and 37.77, respectively. The proportions of these in the total cancer burden for all risk factors combined decreased slightly from 1990 to 2003 and then increased from 2004 to 2019. In 2019, >50% of the cancer burden was concentrated in men aged 55–75 years and women aged 50–70 years. Between 1990 and 2019, there was an increase in age-standardized rates of death, DALYs, YLDs and YLLs among those aged ≥70 years. The age-standardized YLDs rate attributable to secondhand smoke was higher among women; it decreased in men but increased in women, causing a wider gap between the sexes. A greater cancer burden was mainly seen in North America in 1990 and Europe in 2019. Reductions in the annual rate change of cancer burden were found mainly in North America and Oceania, while increases were found in Africa and Asia. In 2019, high–middle- and middle-SDI countries had higher age-standardized rates of deaths, DALYs, YLDs and YLLs than the global level. During 1990 and 2019, the largest decline in cancer burden was seen in high-SDI countries, while middle- or low-SDI countries experienced increases in all age-standardized rates.

**Conclusions:**

Cancer burden attributable to secondhand smoke is concerning given the increasing health loss and differences in distribution of cancer burden worldwide. Further studies are needed to investigate the causes of disparities in cancer burden attributable to secondhand smoke and to improve understanding of the contribution of secondhand smoke to the burden of different types of cancer.

## 1 Introduction

Cancer is widely regarded as a global health threat that contributed to ~10 million deaths in 2020 and nearly one in six deaths (1). It is estimated that the annual number of new cancer cases will increase to 21.6 million by 2030 (2), causing a major global burden that varies markedly across countries and territories. New cases and deaths from cancer have been substantially increasing in low- and middle-income countries because of aging and increased prevalence of other risk factors, and >70% of cancer deaths are expected to occur in Africa by 2030 (3).

Exposure to modifiable risks, such as tobacco use, alcohol consumption, unhealthy diet, physical inactivity and air pollution, is directly associated with cancer treatment and prognosis, such as poor therapeutic response rate, increased recurrence, and treatment complications (4). Smoking or tobacco use is well known as a leading cause of cancer and related death, accounting for ~25% of cancer deaths worldwide (3). It is reported that tobacco smoke contains >7,000 chemicals; at least 70 of which are known to cause cancer by damaging DNA (5). Smoking commonly includes active smoking and passive smoking, both of which increase the risk of cancer.

Passive smoking, which is also known as secondhand smoking, significantly increases the risk of many types of cancers, including breast, nasal sinus cavity, and nasopharyngeal cancer in adults, and the risk of leukemia, lymphoma and brain tumors in children (5, 6). According to a meta-analysis, secondhand smoke increased the risk of cancer development by 16% compared with the risk in people who were not exposed to secondhand smoke (5). Some studies showed that secondhand smoke contributed to 1.8% of cancer deaths among men and 50% among women in China (7, 8). This shows that secondhand smoking poses considerable challenges to reducing cancer burden worldwide.

Exploring the risk factors and interventions for cancer has been a primary concern of healthcare providers as well as researchers, yet the cancer burden caused by secondhand smoke is not well understood for the following reasons. First, no study has estimated the global burden of cancer attributable to secondhand smoke, and analyzed the contribution of cancer burden caused by secondhand smoke to the total cancer burden at the global level (9). Second, a meta-analysis was performed to evaluate cancer risk associated with secondhand smoking across all types of cancer (5). However, there was a lack of data related to disability-adjusted life-years (DALYs), years lived with disability (YLDs), and years of life lost (YLLs) of cancer patients exposed to secondhand smoke. Third, studies have mainly focused on lung and breast cancer and secondhand smoking (10–12), or on the influence of parental smoking on the development of pediatric cancer (13, 14), rather than among the whole cancer population. Fourth, several studies were conducted to explore the association of cancer and secondhand smoking in different countries, for example, China (7, 8, 15), Germany (16) and Korea (17). Additionally, disparities in cancer burden attributable to secondhand smoke across sexes, regions and countries with different socioeconomic status cannot be obtained from these studies.

To fill these gaps, this study aimed to investigate the burden of cancer attributable to secondhand smoke by age, sex and Socio-demographic Index (SDI) for 204 countries and territories between 1990 and 2019, drawing data from the Global Health Data Exchange (GHDx) supported by the Institute for Health Metrics and Evaluation (IHME), University of Washington.

## 2 Materials and methods

### 2.1 Data source

The data were extracted from the GHDx database (https://ghdx.healthdata.org), which is a data catalog created and supported by IHME, an independent population health research organization based at the University of Washington School of Medicine. GHDx provides the data and results from the Global Burden of Disease (GBD) studies that have been initiated by IHME since 2002. GBD studies collect and extract data from national and subnational censuses and representative major survey series, such as the Demographic and Health Survey and the Multiple Indicator Cluster Surveys (18). The GBD team of researchers incorporates data from a large number and wide variety of sources to estimate mortality, causes of death and illness, and risk factors. The GHDx includes data for causes, risks, cause–risk attribution, etiology, and impairments, which can be chosen from a selection box and downloaded for further analysis. The data elements recruited in this study included indicators (deaths, YLLs, YLDs and DALYs), locations, age (all ages and age-standardized), sex, cause of cancer (total cancers), and risk (secondhand smoke).

### 2.2 Estimation framework

#### 2.2.1 Geographical units, age groups, and time periods

The data were presented at four levels: global, demographic, regional and SDI. At the global level, the data were analyzed for all ages and both sexes. At the demographic level, the data were evaluated by age and sex. At the regional level, the data were estimated for all ages and both sexes combined for six continents, including Asia, Africa, North America, South America, Europe and Oceania. The SDI is a composite measure to classify socioeconomic development status by measuring lag-distributed income per person, average years of schooling in the population aged >15 years, and total fertility rate in the population aged <25 years. It is a geometric mean of 0 to 1, indicating that a country with a higher SDI would have a higher level of sociodemographic development related to health outcomes (18). At the SDI level, the data were demonstrated according to five predefined SDI groups: high (0.81–1.0), high–middle (0.69–0.81), middle (0.61–0.69), low–middle (0.46–0.61), and low (0–0.46) (18). Estimation of the data was performed every year from 1990 to 2019.

#### 2.2.2 Estimate of secondhand smoke exposure

We applied the definition of secondhand smoke exposure used by GBD 2019, according to which exposure to secondhand smoke referred to current exposure to secondhand tobacco smoke at home, at work, or in other public places (18). Only non-smokers were taken into consideration when estimating secondhand smoke exposure. Non-smokers referred to people who were not daily smokers, including former smokers, occasional smokers, and those who had never been smokers. Both children and adults were evaluated for exposure. The study data on exposure to secondhand smoke analyzed were secondary data from GBD 2019.

#### 2.2.3 Estimate of cancer burden

GHDx provides data on 30 cancer categories that are classified according to the International Statistical Classification of Disease and Related Health Problem, Tenth Revision (ICD-10), including all benign and *in situ* neoplasms (cancer types included in the study can be found in **Supplementary Table 1**). To estimate the total cancer burden attributable to secondhand smoke, the data were used for all types of cancer combined. Indicators measuring cancer burden were deaths, DALYs, YLDs and YLLs. DALYs were the sum of YLDs and YLLs. YLDs referred to the number of life years a person lived with disability caused by cancer. YLLs referred to the number of life years a person lost as a result of dying early from cancer (19).

### 2.3 Statistical analysis

The numbers and rates of deaths, DALYs, YLDs and YLLs were used to describe the status quo and trends in cancer burden. All the numbers were expressed as absolute numbers, while all the rates were expressed as age-standardized rates (per 100,000 people) based on the GBD reference population. To examine the temporal trends, we estimated the percentage change in the age-standardized rates from 1990 to 2004 (the first half of the study period) and 2005 to 2019 (the second half of the study period). A nonparametric test was used to determine if there were significant differences, while the Mann–Whitney U test was used for unpaired groups, and the Kruskal–Wallis H test for more than two groups. The 95% uncertainty interval (UI) was reported for all estimates. Analyses were completed using SPSS version 27.0 (IBM, Armonk, NY, USA). P<0.05 was considered statistically significant.

## 3 Results

### 3.1 Cancer burden attributable to secondhand smoke at the global level

Secondhand smoke moved from the 11th leading risk of cancer DALYs in 1990 to 10th place in 2019. For those younger than 70 years, secondhand smoke was among the top 10 risk factors for cancer DALYs. Compared with those in 1990, most age groups (25–34 and ≥50 years) in 2019 experienced an increase in rank (**Supplementary Table 2**).

**Figure 1:**
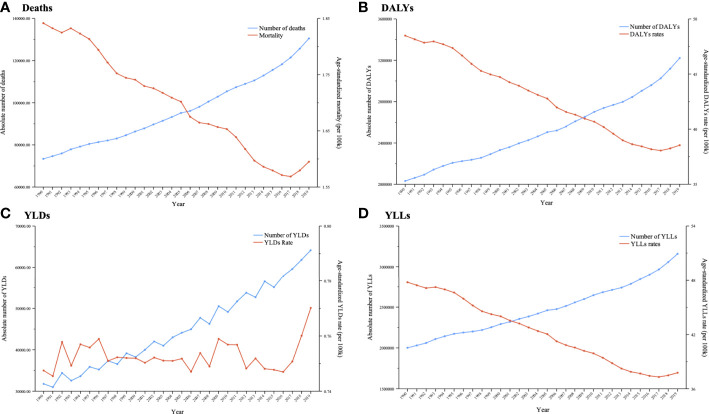
Trends in global cancer burden attributable to secondhand smoke for both sexes combined from 1990 to 2019. **(A)** Deaths; **(B)** DALYs; **(C)** YLDs; **(D)** YLLs.

There were 0.13 million (95% UI: 0.08–0.19) deaths among cancer patients worldwide caused by secondhand smoke and 3.22 million (95% UI: 2.07–4.63) DALYs, of which, 2% came from YLDs and 98% from YLLs in 2019 (**Table 1**). Between 1990 and 2019, the absolute numbers of deaths, DALYs, YLDs and YLLs significantly increased by 77.83% (95% UI: 59.34%–96.49%, P<0.001), 58.48% (95% UI: 42.41%–75.43%, P<0.001), 107.01% (95% UI: 85.38%–128.01%, P<0.001) and 57.73% (95% UI: 41.75%–74.92%, P<0.001), respectively. However, age-standardized rates of cancer-related deaths, DALYs and YLLs attributable to secondhand smoke were significantly reduced from 1.84 to 1.60 (P<0.001), from 48.49 to 38.54 (P<0.001), and from 47.74 to 37.77 (P<0.001), respectively (**Table 1**). For the annual changes in absolute numbers and age-standardized rates, the absolute numbers of deaths, DALYs and YLLs increased consecutively, whereas the age-standardized rates significantly decreased, except for YLDs, between 1990 and 2019 (**Figure 1**).

**Table 1:** Global cancer burden attributable to secondhand smoke for both sexes combined in 1990 and 2019.

	Absolute number		Percentage change in absolute number (%)	*p*	Age-standardized rate		Percentage change in age-standardized rate (%)	*p*
	1990	2019			1990	2019		
Deaths	73382.33 (48515.13, 105076.31)	130497.72 (82600.70, 189503.61)	77.83 (59.34, 96.49)	<0.001	1.84 (2.63, 1.21)	1.60 (1.01, 2.32)	-13.38 (-22.26, -4.32)	<0.001
YLDs	30985.71 (17086.07, 48417.67)	64144.93 (33735.98, 100385.84)	107.01 (85.38, 128.01)	<0.001	0.75 (1.16, 0.42)	0.77 (0.41, 1.20)	3.03 (-7.89, 13.87)	0.31
YLLs	2001729.69 (1332659.92, 2856719.97)	3157378.26 (2016778.65, 4549753.08)	57.73 (41.75, 74.92)	<0.001	47.74 (68.14, 31.79)	37.77 (24.12, 54.49)	-20.88 (-28.92, -12.41)	<0.001
DALYs	2032715.41 (1349038.10, 2888829.75)	3221523.19 (2069953.29, 4632379.11)	58.48 (42.41, 75.43)	<0.001	48.49 (69.09, 32,24)	38.54 (24.77, 55.49)	-20.51 (-28.51, -12.23)	<0.001

1. Data in parentheses are 95% uncertainty intervals; 2. Age-standardized rates (per 100,000 people) based on GBD reference population.

**Figure 2:**
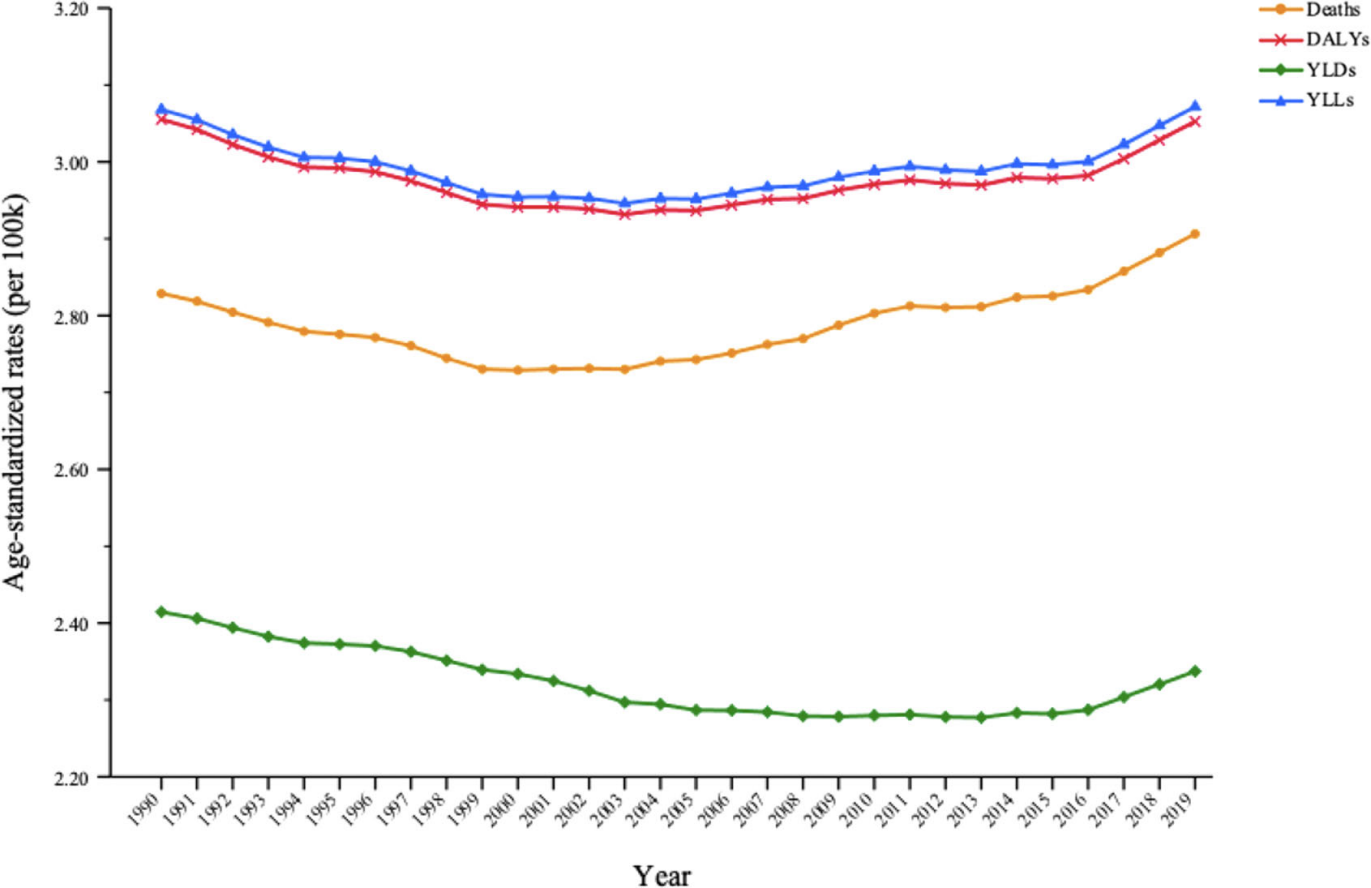
Trends in the proportion of cancer burden attributable to secondhand smoke in total cancer burden caused by all risk factors for both sexes between 1990 and 2019.

Among the total cancer burden for all risk factors combined, secondhand smoke was responsible for 2.91% (95% UI: 2.04%–3.81%) of deaths, 3.05% (95% UI: 2.17%–3.97%) of DALYs, 2.34% (95% UI: 1.68%–2.75%) of YLDs and 3.07% (95% UI: 2.17%–4.0%) of YLLs in 2019. Within the first half of the study (1990–2004), the proportion of cancer burden attributable to secondhand smoke in the total cancer burden for all risk factors combined significantly decreased in terms of cancer deaths, DALYs, YLDs and YLLs. However, during the second half of the study (2005–2019), the proportions of cancer deaths, DALYs and YLLs attributable to secondhand smoke increased significantly (**Table 2**). The proportion of cancer burden caused by secondhand smoke in the total cancer burden that was caused by all risk factors decreased slightly from 1990 to 2003, and then increased from 2004, especially in terms of deaths, DALYs and YLLs (**Figure 2**).

**Table 2:** Contribution of cancer burden caused by secondhand smoke to total cancer burden caused by all risk factors for both sexes between 1990 and 2019.

	Proportion* (%)		Change in proportion (%)					
	1990	2019	1990-2004	*P*	2005-2019	*P*	1990-2019	*P*
Deaths	2.83 (2.02, 3.67)	2.91 (2.04, 3.81)	-3.11 (-5.31, -2.65)	<0.001	5.96 (5.63, 6.83)	<0.001	2.75 (1.20, 3.60)	0.004
YLDs	2.41 (1.84, 2.94)	2.34 (1.68, 2.75)	-4.98 (-6.66, -5.57)	<0.001	2.21 (-2.02, -1.03)	0.01	-3.20 (-8.61, -6.44)	<0.001
YLLs	3.07 (2.20, 3.99)	3.07 (2.17, 4.00)	-3.77 (-5.71, -3.88)	<0.001	4.07 (3.57, 4.51)	<0.001	0.12 (-0.56, 0.33)	0.93
DALYs	3.06 (2.19, 3.97)	3.05 (2.17, 3.97)	-3.85 (-6.36, -4.25)	<0.001	3.95 (3.59, 4.18)	<0.001	-0.09 (-1.15, -0.13)	0.67

Data in parentheses are 95% uncertainty intervals.

*Proportion is calculated by dividing the age-standardized rates of cancer burden attributable to secondhand smoke by the age-standardized rates of total cancer burden caused by all risk factors.

### 3.2 Cancer burden attributable to secondhand smoke by age and sex

For the age-specific distribution of cancer burden attributable to secondhand smoke by sex in 2019, absolute numbers and age-standardized rates of deaths, DALYs and YLLs were higher in women until age 60 years, after which they became higher in men. For cancer deaths, age-standardized mortality increased consecutively with age in both sexes. For DALYs and YLLs, among men, the proportion of absolute numbers was higher in those aged 55–75 years, accounting for 64.73% of DALYs and 53.94% of YLLs. Among women, the proportion of absolute numbers was higher in those aged 50–70 years, accounting for 64.29% of DALYs and 54.01% of YLLs. Absolute YLD number was larger in every age group among women than in men; the age-standardized YLD rate was significantly higher in women aged <75 years (**Supplementary Figure 1**).

**Figure 3:**
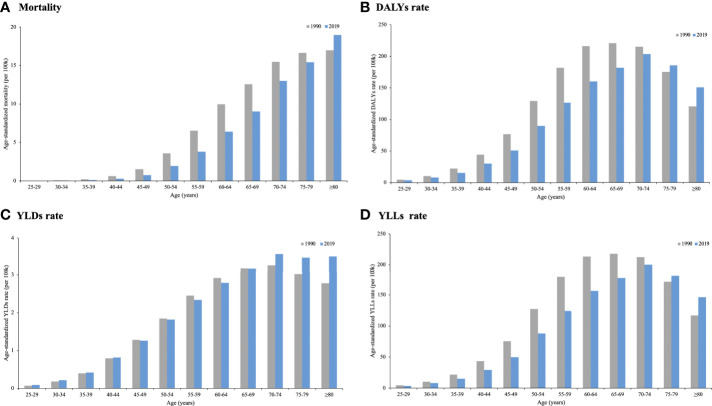
Global cancer burden attributable to secondhand smoke by age in 1990 and 2019. **(A)** Mortality; **(B)** DALYs rate; **(C)** YLDs rate; **(D)** YLLs rate.

In terms of the age differences in global change in cancer burden attributable to secondhand smoke in 1990 and 2019, age-standardized rates of deaths, DALYs, YLDs and YLLs decreased among most age groups. There was an increase in age-standardized mortality among those aged ≥80 years, in the age-standardized DALY rate and YLL rate among those aged ≥75 years, and in the age-standardized YLD rate among those aged ≥70 years (**Figure 3**).

**Figure 4:**
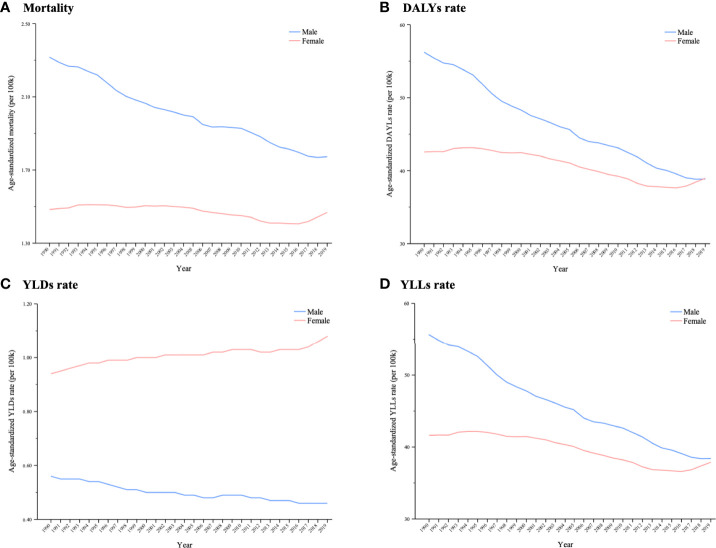
Trends in global cancer burden attributable to secondhand smoke by sex from 1990 to 2019. A **(A)** Mortality; **(B)** DALYs rate; **(C)** YLDs rate; **(D)** YLLs rate.

In 1990, higher age-standardized rates of death, DALYs and YLLs were found in men, and there were considerable differences between men and women. However, the differences in cancer deaths, DALYs and YLLs between men and women decreased over the 1990–2019 period because of a significant reduction among men and a more moderate reduction among women. Moreover, women’s age-standardized DALY rate outpaced that of men in 2019. The age-standardized YLD rate decreased in men from 0.56 (95% UI: 0.32–0.89) to 0.46 (95% UI: 0.25–0.73), but it increased in women from 0.94 (95% UI: 0.39–1.56) to 1.07 (95% UI: 0.44–1.78), causing a wider gap in YLDs between the sexes (**Figure 4**).

### 3.3 Cancer burden attributable to secondhand smoke by geographic region

**Figure 5:**
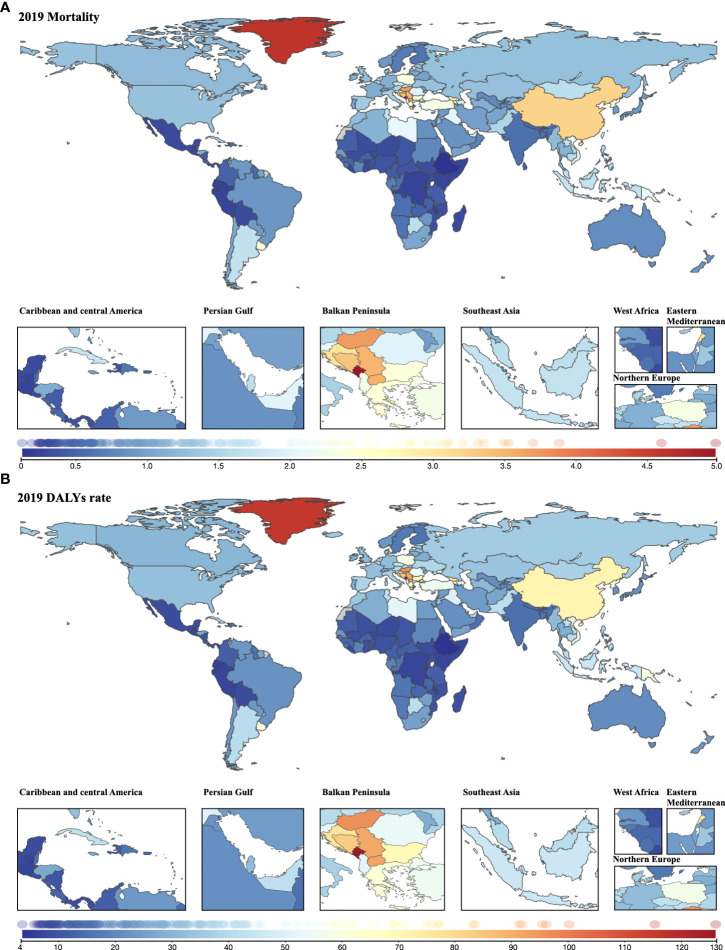
Geographical distribution of cancer burden attributable to secondhand smoke for both sexes combined in 2019. **(A)** Mortality; **(B)** DALYs rate.

In 2019, age-standardized mortality >3.0 was seen in most parts of the Balkan Peninsula, Greenland, and China and Lebanon. A wide range of countries in Africa and South America had <0.50 age-standardized mortalities (**Figure 5A**). The geographical disparities in the age-standardized DALY rate were similar to those in mortality: the highest rate was found in Europe, followed by North America (**Figure 5B**). Among the top 10 countries that had higher mortality rates, nine also had higher DALY rates: Montenegro, Greenland, Palau, Hungary, North Macedonia, Serbia, Bosnia and Herzegovina, Solomon Island, and Lebanon. Geographical distribution of YLDs and YLLs was not different from that of deaths and DALYs, suggesting that most of the countries with the highest rates were also from Europe (**Supplementary Figures 2A, B** **)**. The lowest cancer burden was mainly seen in Africa in terms of deaths, DALYs, YLDs and YLLs.

**Figure 6:**
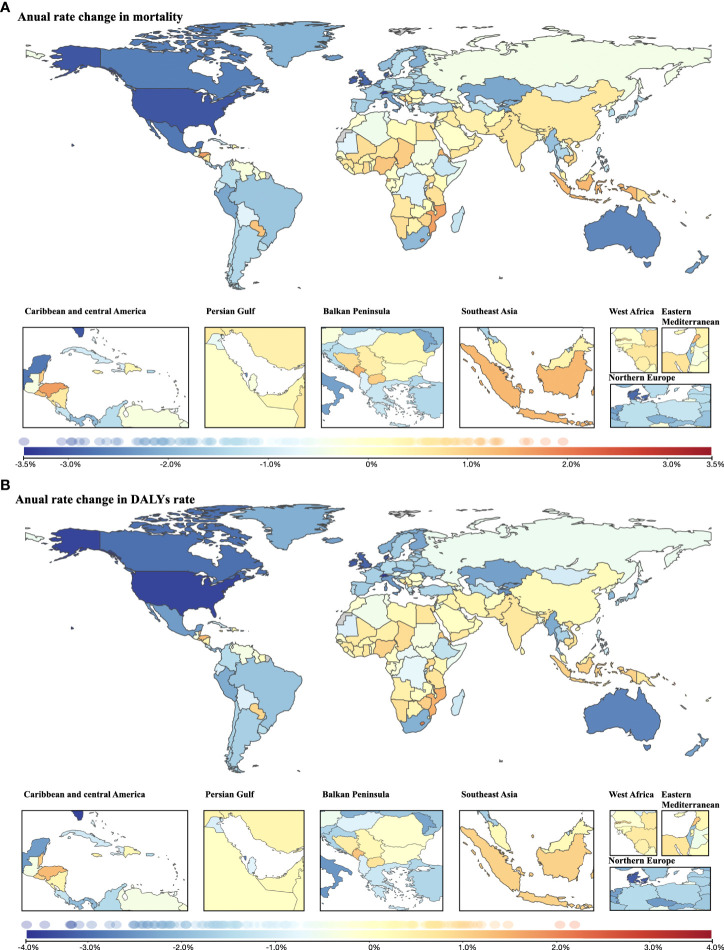
Annual rate change in cancer burden attributable to secondhand smoke for both sexes combined from 1990 to 2019. **(A)** Annual rate change in mortality; **(B)** annual rate change in DALYs rate.

The average annual age-standardized cancer mortality decreased during the three decades in many regions, especially in North America and Oceania, with a reduction of >2.5% per year. Nevertheless, a wide range of countries in Africa, Asia and Europe (mostly in the Balkan Peninsula) experienced increased annual changes in age-standardized mortality (**Figure 6A**). The change pattern for the average annual age-standardized DALY rate remained the same as the pattern for mortality (**Figure 6B**).

In terms of YLDs, the average annual rate decreased mainly in North America and Oceania. In contrast, nearly all locations in Africa experienced increased annual change in the age-standardized rate of YLDs, as did many countries in Asia, where the highest increase in annual rate change was found (Lebanon, 2.93%; followed by Saudi Arabia, 2.33%) (**Supplementary Figure 3A**). Similar to the change pattern for cancer mortality and DALYs, reductions in annual age-standardized YLL rate were observed mainly in North America and Oceania, while increases were observed in Africa, Asia and Europe (mostly in the Balkan Peninsula) between 1990 and 2019 (**Supplementary Figure 3B**).

### 3.4 Cancer burden attributable to secondhand smoke by SDI

In 2019, all the rates were lower than the global level in high-SDI countries, except YLDs (0.84 vs. 0.77). In contrast, high–middle-SDI and middle-SDI countries had higher age-standardized rates of cancer deaths, DALYs, YLDs and YLLs than the global level. Low-SDI countries had the lowest age-standardized mortality (0.50, 95% UI: 0.30–0.74), DALY rate (13.49, 95% UI: 7.69–20.17), YLD rate (0.23, 95% UI: 0.10–0.38) and YLL rate (13.27, 95% UI: 7.58–19.81) in 2019 (**Table 3**).

**Table 3:** Cancer burden attributable to secondhand smoke in 5 SDI groups for both sexes combined in 1990 and 2019 and percentage change from 1990 to 2019.

	Age-standardized rate		Percentage change 1990-2019 (%)	*P*
	1990	2019		
**Deaths**				<0.001
Global	1.84 (1.21, 2.63)	1.60 (1.01, 2.32)	-13.38 (-22.26, -4.32)	
High SDI	2.29 (1.48, 3.29)	1.22 (0.76, 1.79)	-46.83 (-49.97, -43.96)	
High-middle SDI	2.40 (1.57, 3.43)	2.13 (1.36, 3.02)	-11.25 (-21.91, -0.09)	
Middle SDI	1.75 (1.13, 2.53)	2.02 (1.27, 2.96)	15.90 (-2.28, 36.75)	
Low-middle SDI	0.88 (0.55, 1.27)	0.95 (0.60, 1.39)	8.87 (-6.56, 24.23)	
Low SDI	0.46 (0.27, 0.68)	0.50 (0.30, 0.74)	9.66 (-5.59, 25.87)	
**YLDs**				<0.001
Global	0.75 (0.42, 1.16)	0.77 (0.41, 1.20)	3.03 (-7.89, 13.87)	
High SDI	1.17 (0.64, 1.83)	0.84 (0.42, 1.34)	-28.01 (-36.74, -20.33)	
High-middle SDI	0.93 (0.52, 1.44)	1.03 (0.56, 1.6)	11.15 (-1.98, 25.13)	
Middle SDI	0.58 (0.32, 0.91)	0.84 (0.46, 1.31)	45.17 (22.84, 68.69)	
Low-middle SDI	0.32 (0.16, 0.51)	0.42 (0.20, 0.68)	31.96 (11.09, 50.02)	
Low SDI	0.18 (0.08, 0.29)	0.23 (0.10, 0.38)	28.99 (7.92, 50.32)	
**YLLs**				<0.001
Global	47.74 (31.79, 68.14)	37.77 (24.12, 54.49)	-20.88 (-28.92, -12.41)	
High SDI	61.15 (39.98, 87.11)	29.68 (18.89, 42.86)	-51.46 (-54.05, -49.04)	
High-middle SDI	63.09 (41.66, 89.96)	50.25 (32.36, 72.78)	-20.35 (-29.79, -10.18)	
Middle SDI	44.31 (28.36, 63.80)	46.13 (29.41, 67.14)	4.12 (-12.00, 23.13)	
Low-middle SDI	22.82 (14.4, 33.01)	24.02 (15.10, 34.69)	5.30 (-9.71, 20.09)	
Low SDI	12.04 (6.88, 17.96)	13.27 (7.58, 19.81)	10.16 (-5.95, 26.40)	
**DALYs**				<0.001
Global	48.49 (32.24, 69.09)	38.54 (24.77, 55.49)	-20.51 (-28.51, -12.23)	
High SDI	62.32 (40.76, 88.89)	30.52 (19.48, 43. 84)	-51.02 (-53.70, -48.49)	
High-middle SDI	64.02 (42.49, 91.23)	51.28 (33.07, 74.06)	-19.90 (-29.23, -9.69)	
Middle SDI	44.89 (28.79, 64.61)	46.97 (30.04, 68.18)	4.65 (-11.37, 23.39)	
Low-middle SDI	23.14 (14.59, 33.48)	24.45 (15.47, 35.31)	5.67 (-9.50, 20.36)	
Low SDI	12.22 (6.69, 18.22)	13.49 (7.69, 20.17)	10.43 (-5.43, 26.61)	

1. Data in parentheses are 95% uncertainty intervals. 2. Age-standardized rates (per 100,000 people) based on GBD reference population.

Slight changes occurred in the disparities in cancer burden attributable to secondhand smoke among the five SDI groups within the 30 years (**Table 3**). In 1990, the cancer burden was larger than the global level in countries with high and high–middle SDI in terms of cancer deaths, DALYs, YLDs and YLLs. However, the largest decline in cancer burden caused by secondhand smoke was seen in high-SDI countries between 1990 and 2019, resulting in a lower cancer burden than the global level, except for YLDs. Although reductions in all age-standardized rates also occurred in high–middle-SDI countries, the rates of death, DALYs, YLDs and YLLs were still higher than the global level. For countries with middle or low SDI, the cancer burden caused by secondhand smoke in 2019 increased compared with that in 1990, yet the growth was only significant in YLDs, which increased by 45.17% (95% UI: 22.84%–68.69%, P<0.001), 31.96% (95% UI: 11.09%–50.02%, P<0.001), and 28.99% (95% UI: 7.92%–50.32%, P<0.001) in countries with middle, low–middle and low SDI, respectively (**Table 3**).

**Figure 7:**
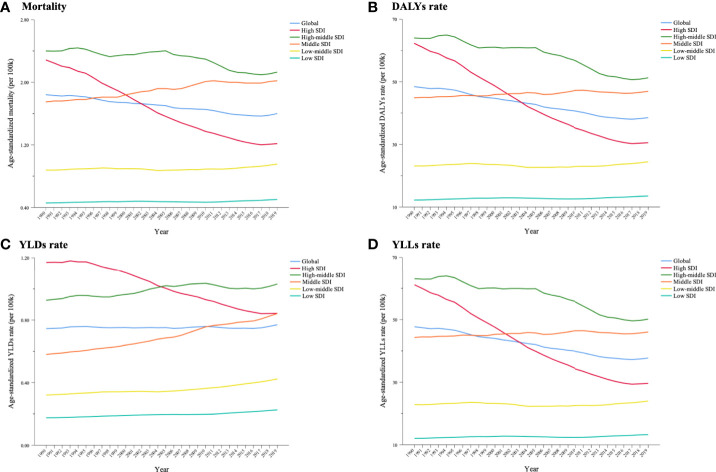
Trends in cancer burden attributable to secondhand smoke in 5 Socio-demographic Index (SDI) groups for both sexes combined from 1990 to 2019. **(A)** Mortality; **(B)** DALYs rate; **(C)** YLDs rate; **(D)** YLLs rate.

From 1990 to 2019, the cancer burden substantially decreased in high-SDI countries. Age-standardized rates of deaths, DALYs, YLDs and YLLs remained modest, climbing in high–middle-SDI countries, and were consistently higher than the global level. The age-standardized mortality, DALY rate and YLL rate in middle-SDI countries surpassed the global level from 1997, while the YLD rate exceeded the global level from 2010 (**Figure 7**).

## 4 Discussion

### 4.1 Principal findings

To our knowledge, this is the first study to estimate the global cancer burden attributable to secondhand smoke. We found that age-standardized rates of cancer deaths, DALYs and YLLs decreased significantly over the past three decades. However, the absolute numbers were consecutively increasing, and the cancer burden caused by secondhand smoke share in relation to the total cancer burden has tended to increase in recent years. In terms of the distribution of cancer burden by demographics, older individuals and women incurred a greater cancer burden attributable to secondhand smoke. There was a significant difference in the status quo and trend of attributable cancer burden across regions. In 2019, the highest cancer burden was seen in Europe, followed by North America and Oceania. Nevertheless, the latter two regions experienced decreases in cancer burden from 1990 to 2019. In contrast, Africa and Asia, which had lower cancer burdens in 2019, experienced increases in the annual changes in deaths, DALYs, YLDs and YLLs. For cancer burden by SDI, high–middle- and middle-SDI countries had higher age-standardized rates of deaths, DALYs, YLDs and YLLs than the global level in 2019. The reduction in cancer burden caused by secondhand smoke was more encouraging in higher-SDI countries. These findings have implications for improvement of population health and reduction of avoidable health loss.

### 4.2 Cancer burden attributable to secondhand smoke at the global level

Secondhand smoke became the 10th leading risk of cancer DALYs. This resulted in increases in the absolute numbers of cancer deaths, DALYs, YLDs and YLLs, particularly in YLDs, which doubled by 2019, and the rising annual proportion of cancer burden caused by secondhand smoke in recent years. Additionally, there were reductions of 13%, 20% and 21% in the rates of deaths, DALYs and YLLs, respectively. These results are consistent with a previous study that investigated cancer burden for all risk factors combined over 30 years (18). This seeming paradox could, to some extent, be explained by the growth of the population and aging. A world population project led by the UN showed that the global population has grown by >1.0% per year, and life expectancy has increased globally from 64.5 years in 1990 to 72.4 years in 2019 (20). As a result, there has been a change in age structure that has given rise to a larger number and proportion of older people worldwide (21). Cancer prevalence is higher in older than in young people; for example, approximately half of the total cancer cases are aged ≥70 years in the United States (US) (22). Therefore, there is an increase in the number of cancer deaths but a decline in cancer mortality.

Furthermore, improvements in access to medical services might lead to an increase in prevalence and disability in cancer survivors who were exposed to secondhand smoke. Thanks to advances in cancer detection and treatments, many patients can be diagnosed at an early stage and receive life-sustaining therapy (23). This is also the reason why there was a dramatic increase in the number of cancer-related YLDs. A previous study documented that the 5-year survival rate for patients with cancer improved for different types of cancer from 1990 to 2009 (22), implying that the number of years that cancer patients live with disability increased as well. Given the increases in absolute numbers and annual contribution of cancer burden attributable to secondhand smoke, there are challenges for healthcare systems that have not been well prepared for population growth and aging (24). We recommend optimization of medical services, such as promoting cancer screening for those at high risk and improving follow-up care for older individuals. In addition, infrastructure should be developed that can meet the needs of the growing number of cancer survivors.

### 4.3 Cancer burden attributable to secondhand smoke by age and sex

In terms of the distribution of attributable cancer burden by demographic characteristics, older individuals incurred a greater burden in 2019. More than half of the cancer burden was concentrated in men aged 55–75 years and in women aged 50–70 years. The age-standardized rates of deaths, DALYs, YLDs and YLLs rose with age for both sexes. As mentioned above, a greater cancer burden in older individuals could be traced to population growth and aging. Additionally, older people are vulnerable to secondhand smoke exposure and have a cumulative risk of exposure to secondhand smoke, so they are more affected by tobacco smoke after they have cancer. Also, older people commonly have other morbidities that exacerbate cancer prognosis (25). According to a report by the US Centers for Disease Control and Prevention, ~80% of adults aged ≥65 years have at least one chronic condition, and 50% have at least two chronic conditions (26). Chronic conditions can cause deterioration of the health loss caused by cancer.

Although there were declines in cancer mortality, DALYs, YLDs and YLLs rates among other age groups in 2019 compared with those rates in 1990, older individuals experienced increases in cancer burden. Similarly, a study that investigated disease burden for all diseases combined revealed that age-standardized all-cause YLD rates were higher in older age (9). As discussed, the proportion of older individuals was higher in 2019 than in 1990 because of the aging population (21). Advances in cancer detection and treatments, and improvements in access to medical services might have led to the increase in prevalence and disability in cancer survivors who were exposed to secondhand smoke. Additionally, it is more likely for them to develop other NCDs because of changes in lifestyle behaviors, such as poor physical activity, which also increase cancer risk and trigger a greater disease burden (23). After cancer occurs in older people, they may be more fragile than younger people, and their health might deteriorate rapidly (25). The increasing burden in the older population has important implications for health policy, including protecting older people from carcinogenic risks, ensuring earlier cancer screening, and providing long-term supportive care for cancer (25).

In terms of sex differences, the gap in cancer burden attributable to secondhand smoke narrowed, resulting from a substantial decline among men and a slight increase among women. Notably, the age-standardized YLD rate continued to rise among women, which was contrary to the situation in men. This pattern reflects that the efforts toward smoking restrictions and cancer control do not equally bring the same benefits to men and women. However, women are more likely to be exposed to secondhand smoke and have an ~30% higher risk of associated cancer (5). To some extent, this implies that the effect of health policies on women is not as favorable as that on men, potentially reflecting inequalities in cancer prevention, intervention and treatment between the sexes over the past 30 years (9). The underlying reasons for this inequality might be complex. Further studies are needed to explore the sex-specific distribution of attributable cancer burden and the disparity of cancer-related health policies in men and women.

Notably, women aged <75 years had a significantly higher YLD rate than men of the same age had in 2019, suggesting that health loss due to cancer is more common in women. One explanation for this is that women might be more attentive to disorders so that they actively seek treatment. In this sense, lifespan with cancer disability might be longer for women. Sex differences in cancer-related health loss should be further examined in future studies.

### 4.4 Cancer burden attributable to secondhand smoke by geographic region

Most of the cancer burden attributable to secondhand smoke was in Europe in 2019, particularly in the Balkan Peninsula. The Balkan Peninsula also had a greater annual rate change in terms of cancer deaths, DALYs, YLDs and YLLs. This finding is consistent with previous studies (18, 23). It is probably driven by high tobacco prevalence, high cancer incidence and rapid aging. According to a WHO report, the prevalence rate of current tobacco use in Europe is nearly as high as the global level and just second to the rate in Southeast Asia in 2019; a slower prevalence rate of decline was seen in Europe from 2000 to 2019 (27). More than 20% of all-cause DALYs were attributable to tobacco use in the Balkan Peninsula in 2019, and >10% of DALYs were attributable to tobacco use in most other European countries (18). Europe has a higher cancer incidence for both sexes combined, and cancers with a high disease burden are more common in Europe (23). For example, the incidence rate and mortality of breast cancer in Europe are higher than those in other regions, while women are the main population that is vulnerable to breast cancer as well as secondhand smoke. The UN project revealed that the European population is growing at a slow rate of 0.3%, but aging is rapid in most European countries (20), in which the proportion of adults aged >65 years will reach 24.3% by 2030 (26). This might also be a reason for the increase in the annual rate change of the attributable cancer burden.

Compared with Europe, some countries in North America and Oceania had higher rates of cancer deaths, DALYs, YLDs and YLLs in 2019, yet the annual rate of change in cancer burden decreased mainly in these two regions. The greater cancer burden caused by secondhand smoke in 2019 might be explained by the relatively high tobacco use and cancer incidence. The tobacco prevalence in North America and Oceania was lower than that in Europe in 2019, resulting in only 6%–8% of all-cause DALYs being attributable to tobacco use in both regions, but it was still higher than that in other regions (18, 27). Cancer incidence was also high in North America and Oceania (23); for example, the breast cancer incidence rate was 91.6 per 100,000 in North America and 85.8 per 100,000 in Oceania. Consistent with the global trends in population growth, the aging population is increasing in these regions as well (20). As a result, the attributable cancer burden in North America and Oceania is not as high as that in Europe but is still higher than that in the other regions. The reduction in the annual change in cancer burden in North America and Oceania may be associated with a decline in smoking prevalence and optimization of cancer care. In western countries, such as the USA, cancer mortality is decreasing, particularly for cancer highly associated with tobacco use, because the tobacco epidemic started the earliest and peaked around the middle of the last century (28–30). Additionally, there have been considerable achievements in cancer treatment since the last century, especially in western developed countries, which have improved the clinical outcomes of cancer patients and prolonged their lifetimes. For instance, in the USA, the total 5-year survival rate for breast cancer increased by >15% from 1975 to 2009, and the overall 5-year survival rate for prostate cancer increased by >30% over the same interval (22). Therefore, secondhand-smoke-related cancer burden has tended to decrease annually.

Africa had lower rates of cancer deaths, DALYs, YLDs and YLLs than other regions had in 2019, yet there was an increase in annual rate change in most African countries. An explanation for this pattern lies in the lower degree of cancer burden attributable to secondhand smoke at baseline in Africa. Thus, although the cancer burden is rising annually in Africa, it still had a lower cancer burden than other regions had in 2019. Furthermore, tobacco prevalence has been consistently lower in Africa than in other regions over the past twelve years (27). Nevertheless, because of the increasing growth in population, which doubled from 1990 to 2019 with an average annual rate of >2.4% (19), African countries experienced an increase in the annual change of cancer related to secondhand smoke. Poor medical treatment and limited access to well-integrated survivor care are also triggers for the rising trends in cancer burden in Africa (31). To avoid continuing growth in cancer burden attributable to secondhand smoke, there is a need for urgent action to improve health of older people and promote medical services for cancer patients (22, 25).

A rising annual cancer burden was also seen in many Asian countries, among which, China stood out and had a greater cancer burden attributable to secondhand smoke than other Asian countries had in 2019. This finding might stem from several aspects. First, all DALYs caused by smoking is high in Asia. According to the GBD Study 2019, in many parts of Asia, tobacco exposure was attributed to 10%–20% of DALYs for all causes combined, while in Liaoning and Heilongjiang Provinces in China, attributable DALYs accounted for >20%, which was higher than in other locations (18). Second, during the past twelve years, there was a large reduction in tobacco prevalence, which fell by >10%, yet the average prevalence (25%) was still higher in the Asian region (27). In particular, the overall smoking rate among adults aged ≥15 years was 26.6% in China in 2018; 50.5% for men and 2.1% for women. More than 40% of the adults reported being exposed to secondhand smoke either at home or in other public places (32). Third, Asia is the most populous region, accounting for a considerable percentage of the aging population worldwide (20). It was projected that adults aged >65 years will comprise 12% of the population in Asia by 2030. China, as the most populous country, is experiencing a decline in fertility and an increase in the average lifespan, resulting in an acceleration in the change in age structure (21). Lastly, limited medical resources and less mature cancer treatment potentially lead to unsatisfactory clinical outcomes (33). Regardless of medical resource distribution or advanced therapy for cancer patients, there are differences between China and other countries. In summary, health loss due to secondhand smoke is increasing annually in Asia, posing remarkable challenges to individuals’ health and health systems. In this sense, it is recommended to reverse the trends through sustained efforts, such as high taxation, smoking advertisement bans, and improvement in cancer screening and treatment (9, 18).

### 4.5 Cancer burden attributable to secondhand smoke by SDI

We found wide disparities in cancer burden attributable to secondhand smoke among the five SDI groups. Although age-standardized rates of cancer deaths, DALYs, YLDs and YLLs were higher in high- and high–middle-SDI countries in 1990, they decreased significantly in high-SDI countries, while they decreased only slightly in high–middle-SDI countries, resulting in a higher cancer burden attributable to secondhand smoke in high–middle-SDI countries compared with the global level. In contrast, the cancer burden attributable to secondhand smoke increased slightly in other SDI countries, especially in middle-SDI countries, which surpassed the global level in later years. That is, the cancer burden decreases more significantly as the SDI level increases, which has also been discovered by previous studies (9, 18, 23). The drivers behind this finding could be complicated because SDI is a composite index.

Smoking is not largely correlated with SDI (18), which indicates that the imbalance in cancer burden primarily stems from differences in socioeconomic development and cancer control across countries. Approximately 50% of cancer cases are seen in high-SDI countries, but only 30% of cancer deaths, 25% of cancer DALYs and 23% of cancer YLLs (9). A higher SDI generally reflects desirable socioeconomic status, which enables cancer patients to receive advanced treatment and incur less health loss (18). However, the greatest increase (52%) in cancer incidence was seen in middle-SDI countries from 2007 to 2017 (9). The cancer burden will shift to less-developed countries because of population growth and aging and the increasing prevalence of risk factors (34).

These findings imply insufficient cancer prevention and treatment across less-developed regions, which demands interventions to address health inequality worldwide (22). To address this international variation, recognizing the interdependencies between socioeconomic status and health is the first step (9). Lower-SDI countries should make more efforts to promote smoking cessation and cancer prevention and control, such as raising tobacco tax, banning smoking in public places, forbidding tobacco advertisement, providing early cancer screening for those at high risk, and improving treatment and follow-up care for cancer patients (23). Additionally, economic support should be provided to the patients for whom cancer treatment expenditure, including for both short-term and long-term care, might be catastrophic.

### 4.6 Limitations

Two limitations of this study should be noted. First, the global burden of cancer attributable to secondhand smoke might be underestimated because data used in this study were directly extracted from the GHDx database, and data for some locations, such as Western Sahara, French Guiana and Svalbard, were not available. This study analyzed the data related to cancer burden attributable to secondhand smoke in most countries or regions of the world. However, there is a need to collect more comprehensive data and conduct further analysis on the cancer burden attributable to secondhand smoke. Second, the disease burden of different types of cancer attributable to secondhand smoke was not assessed in this study. Data on all cancers associated with secondhand smoke are available in the database, whereas the data are not classified according to cancer types. Given the close association between different types of cancer and secondhand smoke, future studies are needed to explore disease burden caused by secondhand smoke according to cancer type.

## 5 Conclusion

The rates of cancer mortality, DALYs and YLLs related to secondhand smoke have decreased globally in the past three decades. However, the contribution of cancer burden attributable to secondhand smoke has shown increasing trends since 2004, as have the life years that cancer patients live with disability or life years lost. More attention should be given to older individuals and women, among whom the cancer burden caused by secondhand smoke exposure has increased or decreased slightly. It is advisable to take more effective actions to enforce a ban on smoking to slow the growth of cancer burden, especially for Asian and African countries with a middle or lower SDI. Further studies are needed to investigate the causes of disparities and trends in cancer burden attributable to secondhand smoke worldwide, and to improve our understanding of the contribution of secondhand smoke to the burden of different types of cancer.

## Data availability statement

The original contributions presented in the study are included in the article/**Supplementary Material**. Further inquiries can be directed to the corresponding authors.

## Author contributions

All authors developed the study concept and design. MM contributed to the statistical analysis, data interpretation and manuscript writing. JW and YJ provided overall guidance and critical revision. MW and YL provided technical support for cancer-related study progress and critical comments. Z-JZ developed the study concept and design, and provided critical revision of article for important intellectual content. All authors revised the manuscript and approved the final version.

## Funding

This study is funded by the Clinical Medicine Plus X - Young Scholars Project, Peking University, the Fundamental Research Funds for the Central Universities (No: PKU2022LCXQ008). The study sponsor has no role in study design, data analysis and interpretation of data, the writing of manuscript, or the decision to submit the paper for publication.

## Acknowledgments

We highly appreciate the research team of the Institute for Health Metrics and Evaluation, University of Washington, for providing publicly available data on global health and visualization tools that formed the basis of our study.

## Conflict of interest

The authors declare that the research was conducted in the absence of any commercial or financial relationships that could be construed as a potential conflict of interest.

## Publisher’s note

All claims expressed in this article are solely those of the authors and do not necessarily represent those of their affiliated organizations, or those of the publisher, the editors and the reviewers. Any product that may be evaluated in this article, or claim that may be made by its manufacturer, is not guaranteed or endorsed by the publisher.

## Supplementary material

Supplementary Figure 1 Age-specific global cancer burden attributable to secondhand smoke by sex in 2019. **(A)** Deaths; **(B)** DALYs; **(C)** YLDs; **(D)** YLLs.

Supplementary Figure 2 Geographical distribution of cancer burden attributable to secondhand smoke for both sexes combined in 2019. **(A)** YLDs rate; **(B)** YLLs rate.

Supplementary Figure 3 Annual rate change in cancer burden attributable to secondhand smoke for both sexes combined from 1990 to 2019. **(A)** Annual rate change in YLDs rate; **(B)** annual rate change in YLLs rate.

## Abbreviations

CDC, Centers for Disease Control and Prevention, The United States of America; DALYs, disability-adjusted life-years; GBD, Global Burden of Disease; GHDx, Global Health Data Exchange; HPV, human papillomavirus; IARC, International Agency for Research on Cancer; ICD-10, International Statistical Classification of Disease and Related Health Problem, Tenth Revision; IHME, Institute for Health Metrics and Evaluation; LDI, lag-distributed income per person; NCDs, non-communicable diseases; NCI, National Cancer Institute of America; SDI, Socio-Demographic Index; WHA, World Health Assembly; WHO, World Health Organization; YLDs, years lived with disability; YLLs, years of life lost; UI, uncertainty interval.
