# Supplementary figures and images for: Global trends and regional differences in the burden of cancer attributable to secondhand smoke in 204 countries and territories, 1990–2019
Source: Front Oncol. 2022 Oct 11;12:972627. doi: 10.3389/fonc.2022.972627 (PMC9592919; doi:10.3389/fonc.2022.972627)

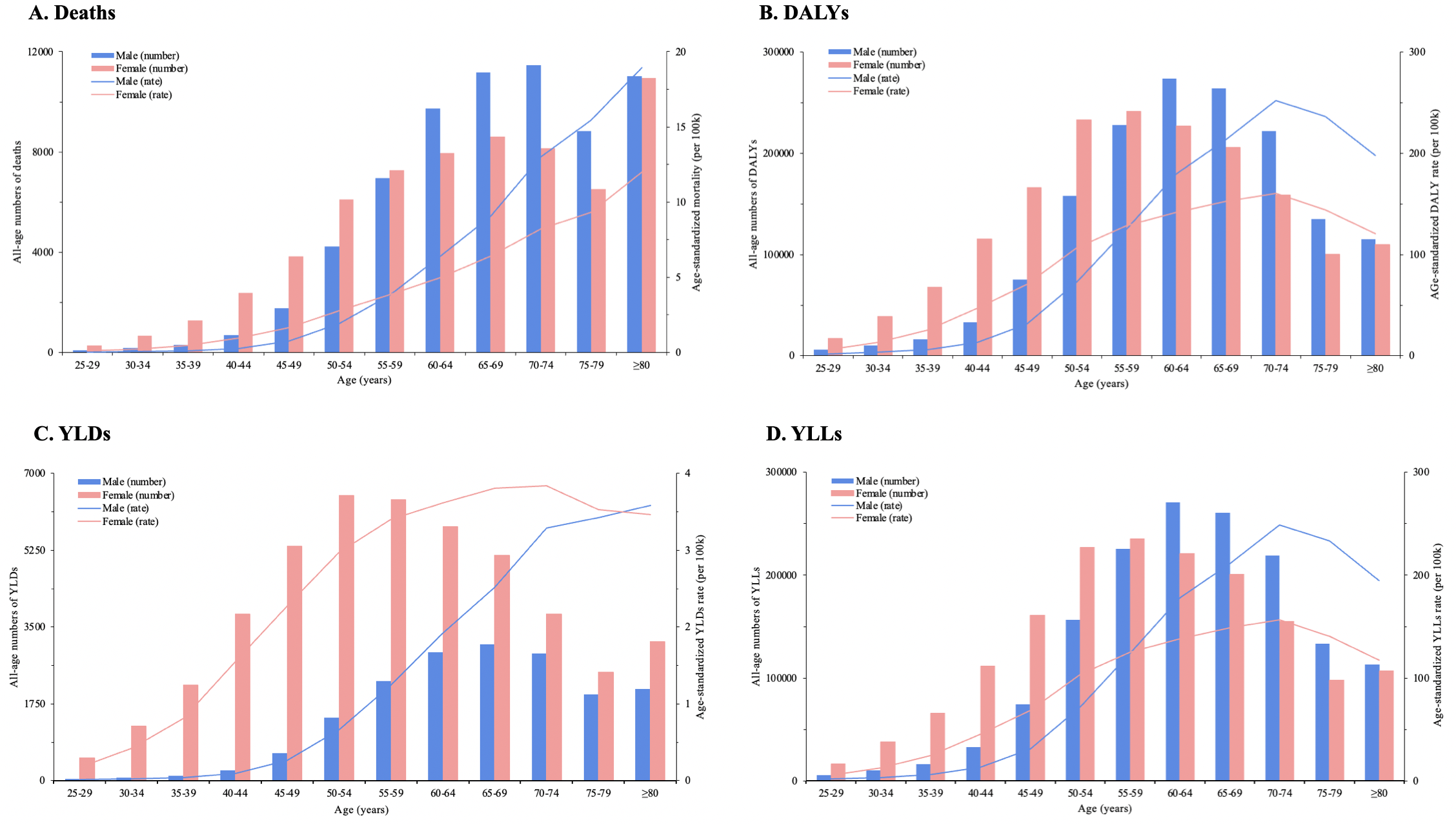

Supplement: Supplementary Figure 1 — Age-specific global cancer burden attributable to secondhand smoke by sex in 2019. (A) Deaths; (B) DALYs; (C) YLDs; (D) YLLs. [file Image_1.jpeg]

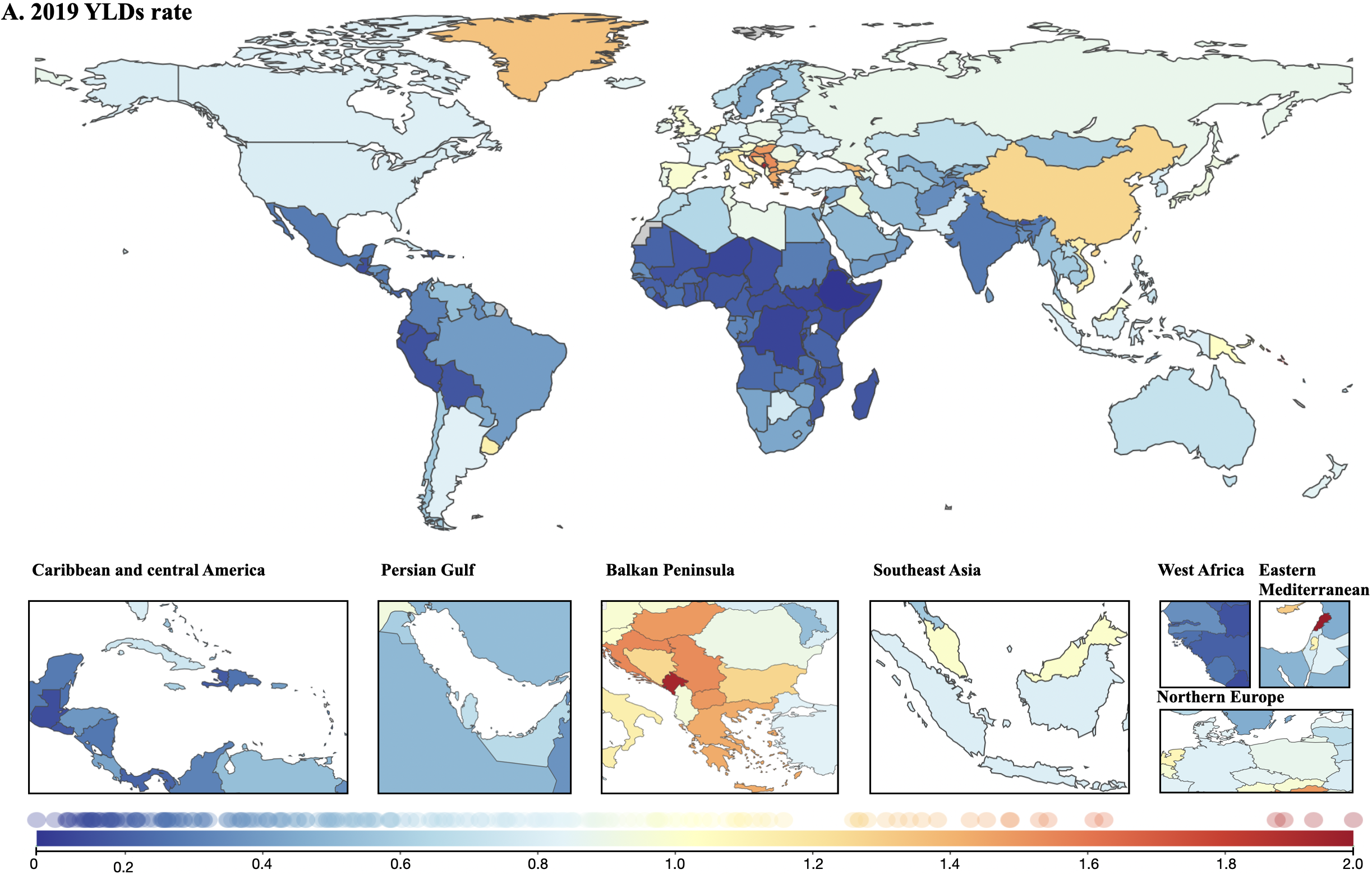

Supplement: Supplementary Figure 2 — Geographical distribution of cancer burden attributable to secondhand smoke for both sexes combined in 2019. (A) YLDs rate; (B) YLLs rate. [file Image_2.jpeg]

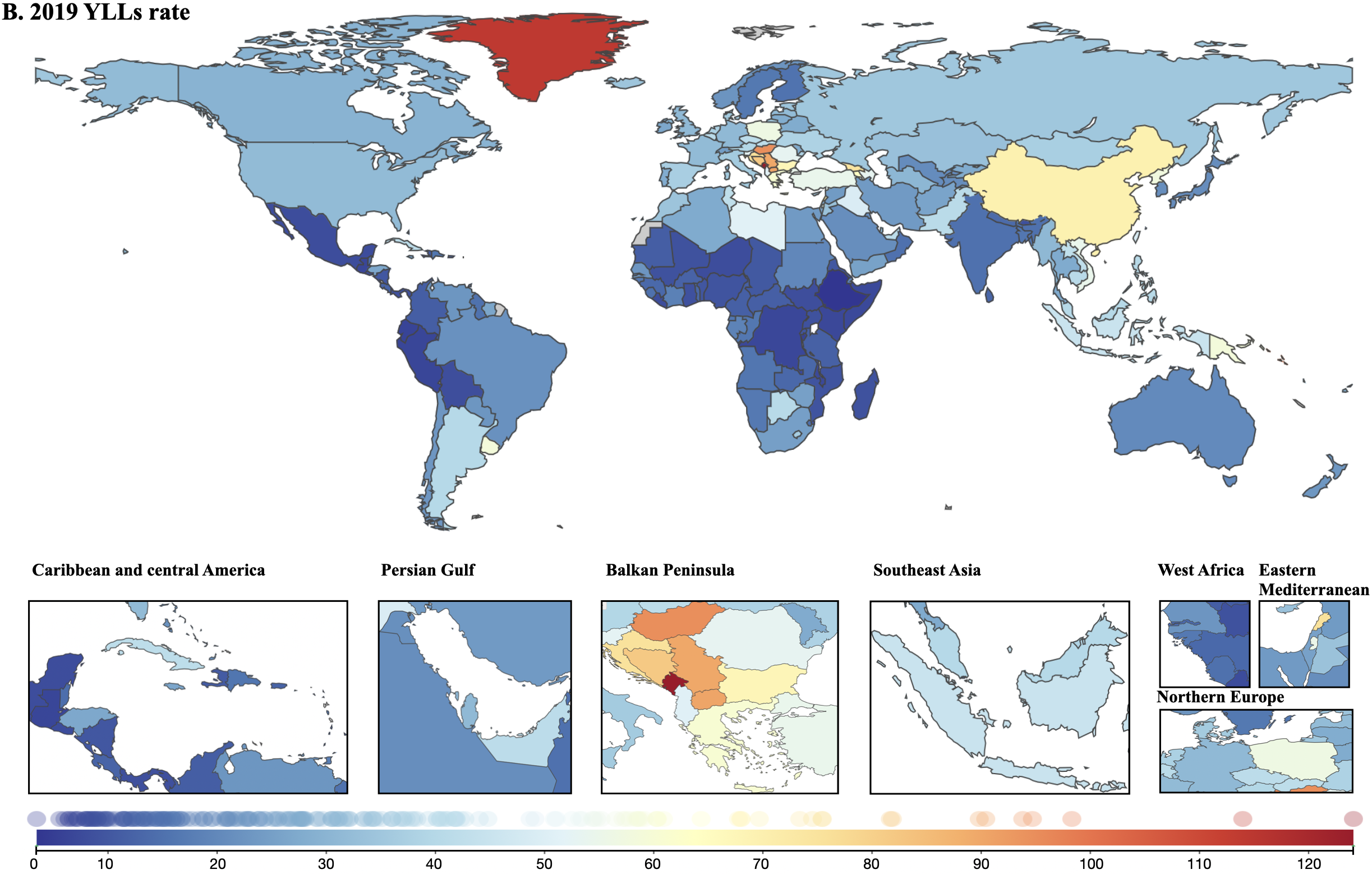

Supplement: Supplementary Figure 3 — Annual rate change in cancer burden attributable to secondhand smoke for both sexes combined from 1990 to 2019. (A) Annual rate change in YLDs rate; (B) annual rate change in YLLs rate. [file Image_3.jpeg]

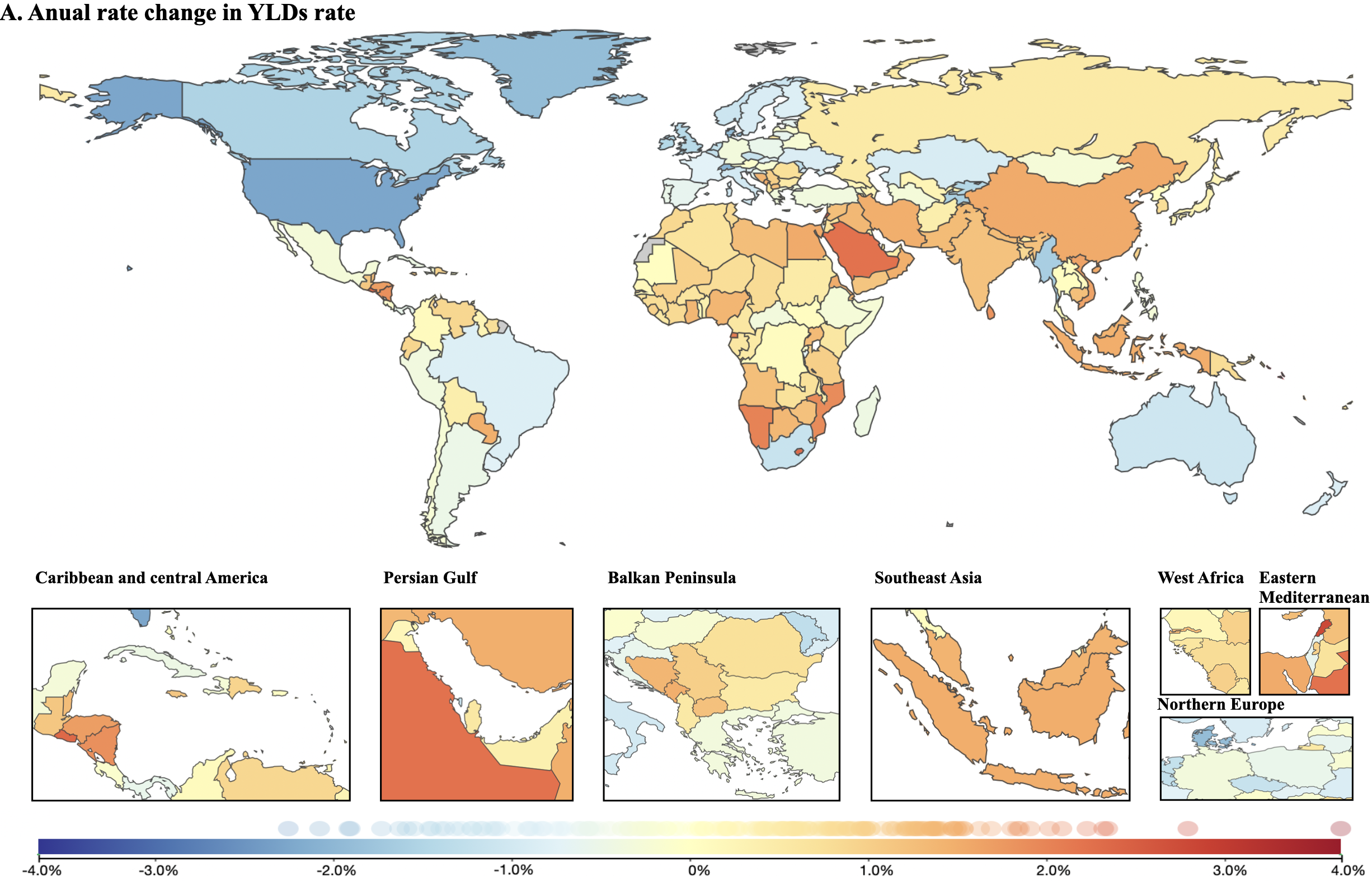

Supplement: Supplementary file 4 [file Image_4.jpeg]

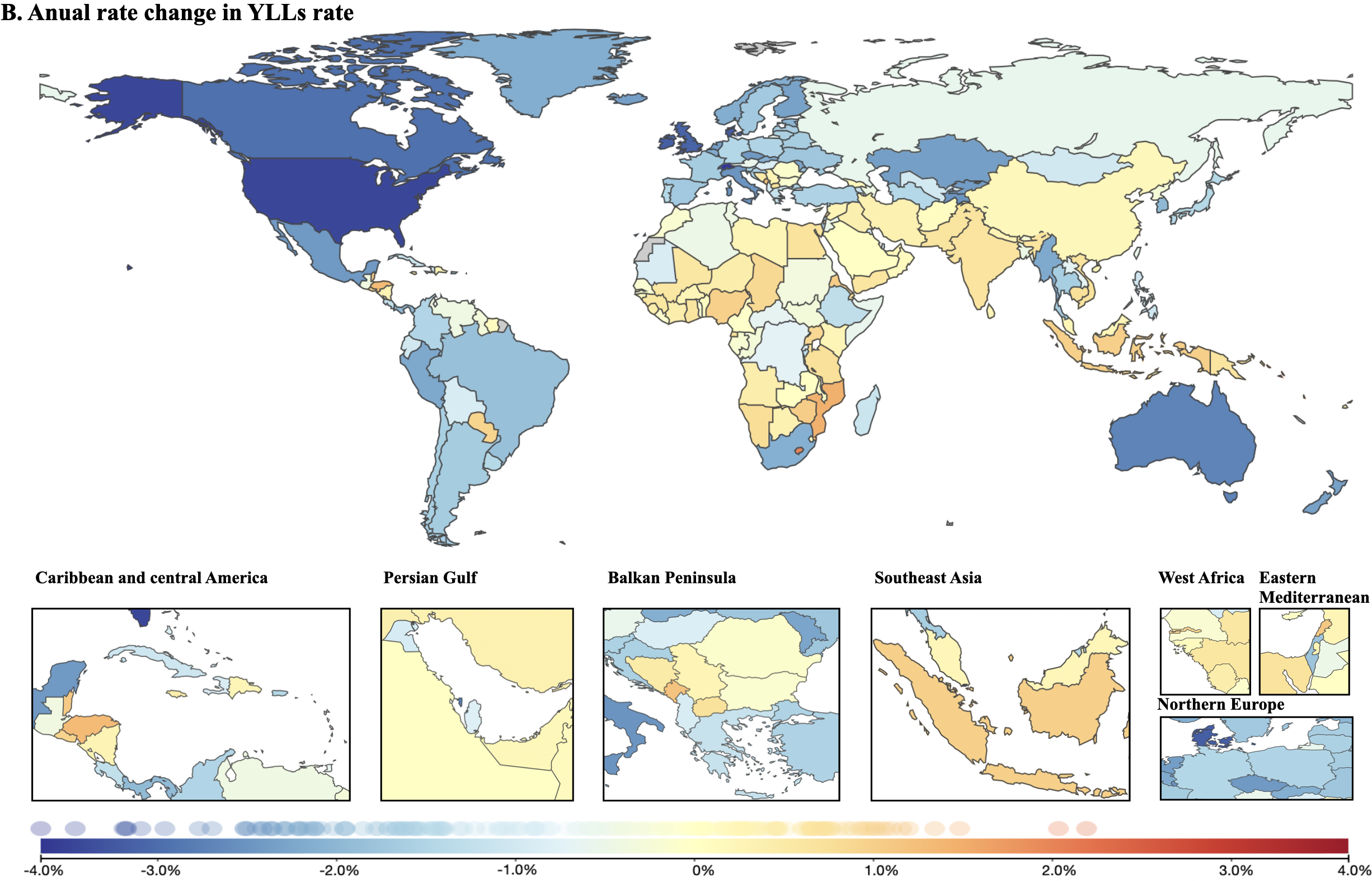

Supplement: Supplementary file 5 [file Image_5.jpeg]
