# Supplementary material for: Global trends and regional differences in the burden of cancer attributable to secondhand smoke in 204 countries and territories, 1990–2019
Source: Front Oncol. 2022 Oct 11;12:972627. doi: 10.3389/fonc.2022.972627 (PMC9592919; doi:10.3389/fonc.2022.972627)
Supplement: Supplementary file 6 [file Table_1.docx]

**S****upplementary Table 1. List of cancer types included in the study**

| **No.** | **Cancer** |  |
| --- | --- | --- |
| **1** | Lip and oral cavity cancer |  |
| **2** | Nasopharynx cancer |  |
| **3** | Other pharynx cancer |  |
| **4** | Esophageal cancer |  |
| **5** | Stomach cancer |  |
| **6** | Colon and rectum cancer |  |
| **7** | Liver cancer | Liver cancer due to hepatitis A |
|  |  | Liver cancer due to hepatitis B |
|  |  | Liver cancer due to alcohol us |
|  |  | Liver cancer due to NASH |
|  |  | Liver cancer due to other causes |
| **8** | Gallbladder and biliary tract cancer |  |
| **9** | Pancreatic cancer |  |
| **10** | Larynx cancer |  |
| **11** | Tracheal, bronchus, and lung cancer |  |
| **12** | Malignant skin melanoma |  |
| **13** | Non-melanoma skin cancer | Squamous cell carcinoma |
|  |  | Basal cell carcinoma |
| **14** | Breast cancer |  |
| **15** | Cervical cancer |  |
| **16** | Uterine cancer |  |
| **17** | Ovarian cancer |  |
| **18** | Prostate cancer |  |
| **19** | Testicular cancer |  |
| **20** | Kidney cancer |  |
| **21** | Bladder cancer |  |
| **22** | Brain and central nervous system cancer |  |
| **23** | Thyroid cancer |  |
| **24** | Mesothelioma |  |
| **25** | Hodgkin lymphoma |  |
| **26** | Non-Hodgkin lymphoma |  |
| **27** | Multiple myeloma |  |
| **28** | Leukemia | Acute lymphoid leukemia |
|  |  | Chronic lymphoid leukemia |
|  |  | Acute myeloid leukemia |
|  |  | Chronic myeloid leukemia |
|  |  | Other leukemia |
| **29** | Other malignant neoplasms |  |
| **30** | Other neoplasms | Myelodysplastic, myeloproliferative, and other hematopoietic neoplasms |
|  |  | Benign and in situ intestinal neoplasms |
|  |  | Benign and in situ cervical and uterine neoplasms |
|  |  | Other benign and in situ neoplasms |
