# Supplementary material for: Global trends and regional differences in the burden of cancer attributable to secondhand smoke in 204 countries and territories, 1990–2019
Source: Front Oncol. 2022 Oct 11;12:972627. doi: 10.3389/fonc.2022.972627 (PMC9592919; doi:10.3389/fonc.2022.972627)
Supplement: Supplementary file 7 [file Table_2.docx]

Supplementary Material

# Supplementary Table

**Supplementary Table 2.** Ranking of age-specific global DALYs for all cancers attributable to secondhand smoke in 1990 and 2019

|  | 1990 Rank | 2019 Rank | Change of rank |
| --- | --- | --- | --- |
| Age-standardized | 11 | 10 | +1 |
| 25-29 | 9 | 8 | +1 |
| 30-34 | 10 | 9 | +1 |
| 35-39 | 8 | 9 | -1 |
| 40-44 | 8 | 10 | -2 |
| 45-49 | 8 | 9 | -1 |
| 50-54 | 8 | 7 | +1 |
| 55-59 | 9 | 7 | +2 |
| 60-64 | 10 | 7 | +3 |
| 65-69 | 11 | 10 | +1 |
| 70-74 | 12 | 11 | +1 |
| 75-79 | 14 | 12 | +2 |
| ≥80 | 16 | 13 | +3 |
